# Geospatial and econometric approaches or older driver safety: Analysis of crash injury severity of regional highways

**DOI:** 10.1371/journal.pone.0307927

**Published:** 2025-01-27

**Authors:** Daiquan Xiao, Dajie Zuo, Xuecai Xu, Quan Yuan

**Affiliations:** 1 School of Civil and Hydraulic Engineering, Huazhong University of Science and Technology, Wuhan, China; 2 School of Transportation and Logistics, Southwest Jiaotong University, Chengdu, China; 3 State Key Laboratory of Automotive Safety and Energy, School of Vehicle and Mobility, Tsinghua University, Beijing, China; 4 Center for Intelligent Connected Vehicles and Transportation, Tsinghua University, Beijing, China; University of Wisconsin-Milwaukee, UNITED STATES OF AMERICA

## Abstract

This study tried to focus on the older drivers’ group and explore the impact factors of injury severity involving older drivers from geo-spatial analysis. To reach the goal, a spatial analysis was proposed employing geographic information systems (GIS) with a case study application to two counties in Nevada. First, crash clusters were explored using Density-Based Spatial Clustering of Applications with Noise (DBSCAN) approach to investigate the spatial crash pattern for older drivers, and determine high risk locations of injury severity. Next, Bayesian spatial binary probit model was presented in order to determine the significant impact factors of injury severity involving older drivers. It was found that at-fault driver condition and vehicle condition, not-at-fault vehicle action and road factors were significant factors for injury severity of older drivers. Results revealed that DBSCAN provides a solid option for hotspot identification of injury severity and Bayesian spatial binary probit model addresses the factor determinants spatially. The GIS-based spatial analysis can benefit more reliable older driver-concentrated evaluation and injury severity analysis.

## Introduction

In accordance with the standard of United Nation, the region with 7% population aged over 65 years is regarded as ageing society, which is one of the most important social change and challenges in this century. With more and more countries and regions stepping into ageing society, this trend is expected to boost in the coming years, whereas older drivers are considered as one of most vulnerable and the highest risk roadway users in accordance with crash severe injuries and fatalities. Due to the reduction in the sensory, cognitive and decision-making abilities for older drivers, this variation may lead to the rising of crash injury [1]. As predicted, crashes involved older drivers would increase by 178% while fatal crash would increase by 155% from 1999 to 2030 [2]. A through literature examination reveals that there is still a gap by examining the spatial features of crash injury about older drivers as well as determining the significant impact factors of injury severity.

To mitigate and reduce the burden of injury, location-specific vehicle crashes of older drivers can be one way to highlight the injury severity so as to conduct spatial investigations and determine the influencing factors. Based on GIS spatial analysis, the objectives of this study are to identify the clustering of older drivers’ injury, describe the locations over space, and accommodate geo-visualization and geo-statistical factors involved in crash injury so as to further provide potential insights for reducing severe injury and fatality of older drivers.

As for the detailed contents, we firstly proposed DBSCAN approach, and conducted the spatial analysis in two urban counties in Nevada, namely Clark County and Washoe, which ranks the top two of state in crash injuries of older drivers. Followed the spatial analysis, an econometric modeling was put forward to identify the significant influencing factors with a spatial probit model. The results can be helpful for decision makers to understand the spatial properties deeply and determine the main features of crash injury involving older drivers.

## Literature review

There have been a variety of approaches and methods about crash frequency or injury severity, in this study only the studies about old drivers or age-related are considered.

### A. The effect of age on crash injury

There have been a variety of studies about the effect of age on crash-related injuries [1, 3], and some have verified that age groups show different effects on crash injury. For example, Tavris et al. (2001) [4] evaluated age and gender patterns in vehicle crash injuries. The results showed that male drivers losing control crashes exceeded female rates in all age groups, but for passengers’ injury rates were larger for elder females. Similar study by Lam (2002) [5] investigated the relation between distractions and risk of vehicle injury among drivers with different ages. It was found that age affected the relationship between in-vehicle distraction and the risk of vehicle crash injury. Newgard (2008) [6] explored the association between age and serious injury in vehicle crashes. The multivariable regression models displayed that age was a significant predictor of serious injury, but there was no age difference for an older occupant with injury risk. Cheung and McCartt (2011) [7] compared the passenger vehicle-involved per 100,000 licensed drivers for older drivers and middle-age drivers. The results showed that fatal crash-involved rates decreased for older and middle-age drivers. Cicchino and McCartt (2014) [8] and Cicchino (2015) [9] gave the reason that fatality rates among older drivers reduced. Contrarily, Kim et al. (2013) [10] found that older drivers revealed a higher probability of a fatal injury, and showed age and gender-based population heterogeneity. Similar study by Carter et al. (2014) [11], Russo et al. (2014) [12], Braitman et al. (2014) [13], and Palumbo et al. (2019) [14] verified the findings.

From the perspective of health care costs, Shen and Neyens (2015) [15] checked out the relationships between drivers’ age, gender and crash types. Hierarchical linear regression models were constructed and the results displayed that older male drivers were required higher health care costs. About the trends in the crash involvement of older drivers, Thompson et al. (2018) [16] determined the increasing or decreasing factors, in which population and license were increased while the total crashes, serious injuries and fatalities were kept stable. Kim and Ulfarsson (2019) [17] compared the older adult pedestrians with younger ones by employing random-effects logistic regression models. The results revealed that older adults were more involved in pedestrian crashes and severe injury. Ayuso et al. (2020) [18] focused on the effect of older drivers in crash severity. Parametric and semi-parametric regression models analysis results determined that crash severity and expected costs of crashes increased with the age of 75 drivers. The study by Gim (2022) [19] analyzed the crash injury severity by older drivers with generalized ordered logit analysis. It was revealed that severity, the time of the day and related variables were significant to fatal and serious injuries, and age and alcohol changed the severity to most severe fata level.

### B. Geospatial approach analysis

Geospatial approach provides a potential insight on the crash analysis. Effati et al. (2015a) [20] proposed a geospatial method based on fuzzy classification and regression tree (FCART) to forecast the crash severity on two-lane & two-way roads. The results revealed that bagged-FCART model performed better the similar terms in accuracy of crash severity. Continuously, Effati et al. (2015b) [21] extended the geospatial approach based on machine learning approaches to investigate crash severity on a regional highway corridor. Support vector machine (SVM) outperformed in addressing spatial dependence and spatial heterogeneity effects. Dezman et al. (2016) [22] investigated hotspots and reasons of motor vehicle crashes with geospatial analysis. Multivariate spatial regression model was employed to assess the impact of socioeconomic indicators on hotspots, and the results indicated geospatial characteristics influenced the risk factors. Recent study by Ouni and Belloumi (2019) [23] examined the pattern of crash hot zones versus probable hot zones with geospatial analysis. The determined hot zones and probable hot zones revealed various regional and temporal features, and spatial autocorrelation indices per region accommodated the diversity within the regions.

Arc GIS is a helpful tool to explore the spatial and temporal features of crash risks. Early study by Li et al. (2007) [24] analyzed intra-city motor vehicle crashes based on GIS. Bayesian approach was employed to determine the spatial-temporal patterns of crash risks, and the results indicated that the approach was effective in estimating crash risks and selecting safer routes for travelers. Dai (2012) [25] extended to pedestrian injuries with spatiotemporal clustering technique in a GIS environment. The logistic regression model was employed to identify the risky factors of pedestrian-vehicle crashes, and age, pedestrian maneuvers, and inadequate lighting were found to be significant impact factors for pedestrian injuries. Similar study by Hanson et al. (2013) [26] verified the findings with Google street view. Focusing on aging-involved crashes, Ulak et al. (2017) [1] investigated effect of spatial dependency with number and percentage of 65+ populations using GIS. The findings revealed that crashes including aging drivers were different from other age group crashes both spatially and temporally. From the perspective of built environment, Huang et al. (2018) [27] explored the spatial relation with crashes using geographically weighted regression model. It was found that commercial use percentage, road mileage percentage and intersection density revealed significance. Similarly, Prato et al. (2019) [28] verified the results of built environment, land use and traffic conditions. The work by Hu et al. (2020) [29] continued the impacts of building environment and road characteristics on pedestrian crashes. Binary logistic regression and tree-based models were combined and the results indicated that pedestrian severity was highly related to lighting conditions, road facilities and pedestrian age and behavior.

Clustering analysis has been widely applied in various fields and has been accepted as one critical data mining approach [30–32]. Currently, there are a variety of clustering algorithms, such as partition-based methods, hierarchical methods, density-based methods, grid-based methods, model-based methods, etc. Among all them, density-based methods due to the ability of solving irregular patterns and noises have been widely employed, in which DBSCAN (Density-Based Spatial Clustering of Applications with Noise) considers density-based clustering algorithm, whose main feature lies in dealing with spatiotemporal data mining [33]. By comparing DBSCAN with K-means, and Quadratic variation algorithms, Dudik et al. (2015) [30] found out that DBSCAN algorithm had a higher sensitivity and performed the best. Therefore, DBSCAN is considered for this work.

### C. Influencing factors analysis of crash injury

In recent years, there have been a number of various approaches and perspectives proposed in crash injury severity [34, 35], among which multivariate regression analysis has been regarded as one critical method dealing with two or more dependent variables with correlation issue. This provides theoretical basis for our study.

As for the older drivers, Lam and Lam (2005) [36] investigated the relation between sudden illness and risk of vehicle crash injury among older drivers. Boufous et al. (2008) [37] examined the impact of environmental, vehicle, crash and driver features on injury severity. Multivariate analysis revealed rurality, complex intersections, speed limit, driver error, speeding and set belt use were significant indicators. Thompson et al. (2013) [16] identified the driver, vehicle and environmental factors related to the crashes of older rural drivers. Logistic regression model analyzed that each factor improved the probability of a serious or fatal injury. From the perspective of at-fault older drivers, Chin and Zhou (2018) [38] identified influencing factors of older drivers in light vehicle crashes in Singapore. Binary logit model analyzed that peak periods, festive seasons, curb lanes, wet surfaces were significant to the older drivers being at fault.

Summarized from the studies above, so far few studies have integrated the injury severity of older drivers with geo-visualization and geo-statistics, and Ulak et al. (2017) [1] made the attempt and provided the potential insight for our study. Therefore, the purpose of this paper is to (a) investigate the crash pattern and high risk locations of older drivers with geo-spatial analysis of crashes, and (b) identify the significant impact factors of the injury severity of older drivers with spatial probit model. The results may contribute to the development of more reliable older driver-concentrated evaluation and injury severity analysis.

## Methodology

### A. Data description

**Fig 1:**
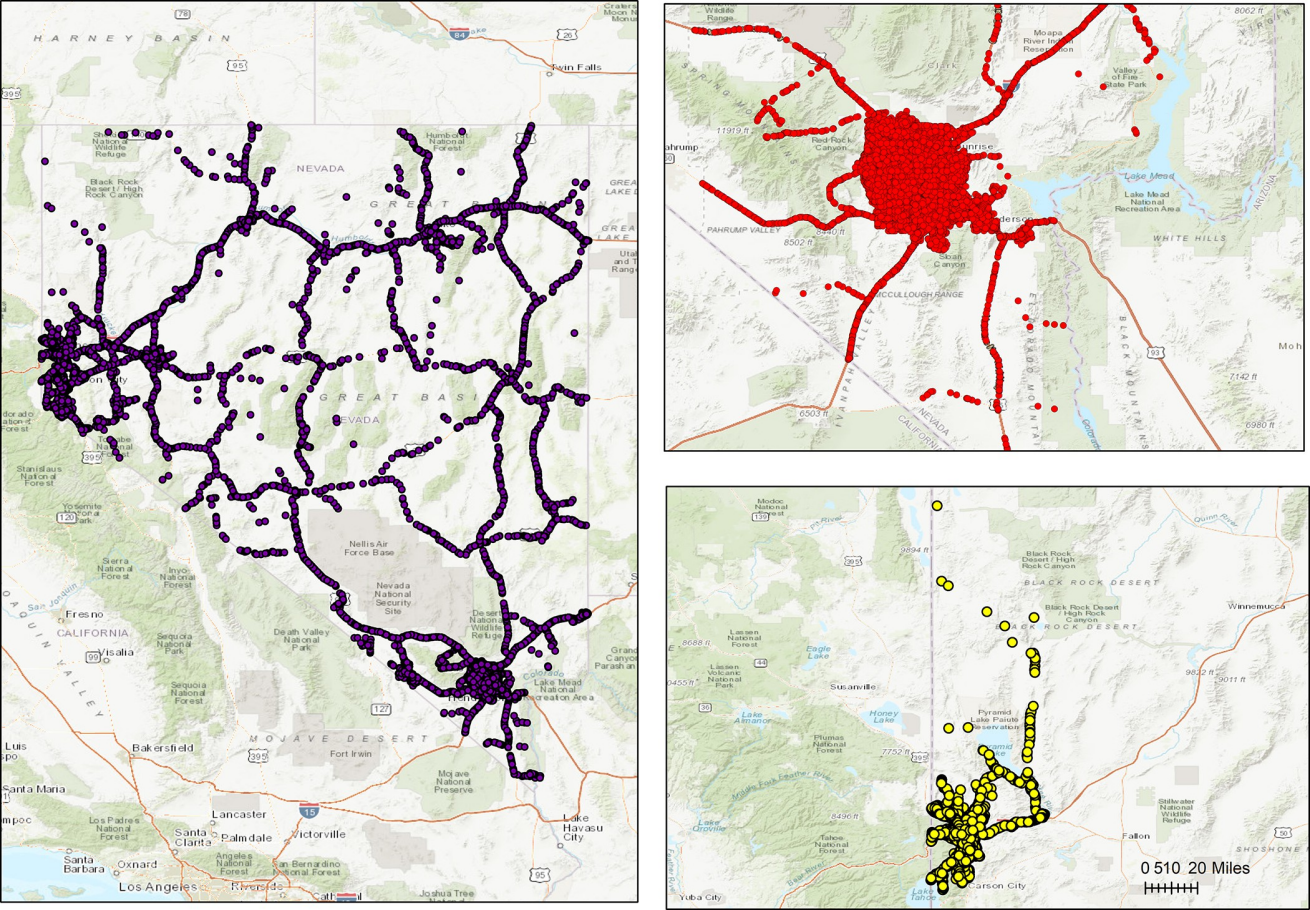
Crash distribution in Nevada from 2014 to 2017. (a) Crash Distribution in Nevada, (b) Crash Distribution in Clark County and Washoe.

The crash data collected from 2014 to 2017 with Arc GIS open data site were kept by Nevada Department of Transportation (NDOT), and 146, 751 crashes (shown in Fig 1(A)) were the target population chosen in this study, including Clark County, Carson City, Washoe, Eureka White Pine, and the rest 12 counties and cities. Among 17 counties and cities, Clark County and Washoe based on the number of crash injuries rank top 2 (as shown in Fig 1(B)), accounting for 108,306 and 23,415, respectively, totally about 90% of all, thus the following analysis is focused on the two counties. In Fig 1, each point represents one crash in the database.

Four main aspects of crashes were extracted and considered as dependent and independent variables: injury-related features, the human-related characteristics, the vehicle-related profiles, roadway-related characteristics and the environment condition.

**Fig 2:**
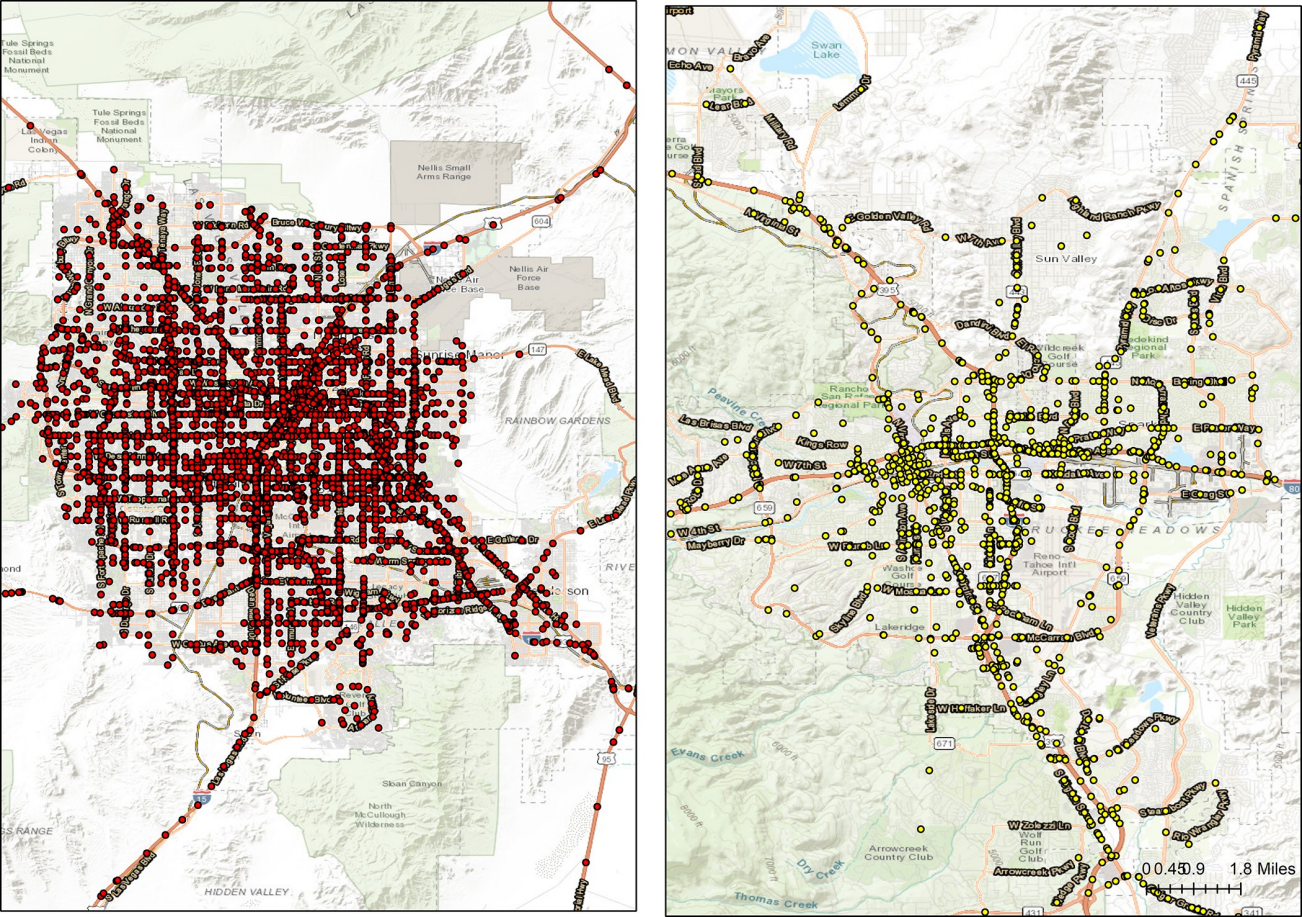
Crashes of older drivers in Clark County and Washoe. (a)Clark County, (b)Washoe.

In accordance with Devlin and McGillivray (2016) [39], drivers aged 65 and more are considered as older drivers. In Nevada, the injury severity is typically classified as property damage only (PDO), injury and fatality. In the sample collected, the fatality only explained for 0.5%, thus merging the injury and fatality categories is not expected to substantially affect the analysis [40]. Therefore, in order to examine the impact factors of injury severity, the dependent variables in the proposed model were considered as binary, in which the response of interest was referred to PDO, and injury and fatality was considered as the contrast, i.e. binary probit model. As shown in Fig 2, crash injuries of older drivers in Clark County and Washoe are 6,670 and 1,640, respectively, and display different spatial features, hence spatial binary probit model was proposed by taking into account of spatial features and binary probit model.

As denoted above, the independent variables here include the driver-related characteristics, the vehicle-related profiles, roadway-related characteristics and the environment condition. According to the classification by NDOT, when the crash occurs, if there are two or more vehicles involved, the at-fault vehicle is considered as vehicle 1, while the not-at-fault vehicles are considered as vehicle 2, in this way the at-fault and not-at-fault older drivers can be separated from the dataset, as well as driver’s conditions (e.g. normal, fatigue, physical impairment, distracted, etc.). Followed this method, the vehicle-related variables include the total vehicles involved, vehicle types, vehicle direction, vehicle action (e.g. changing lanes, making U-turn, passing other vehicles, etc.), and vehicle conditions (e.g. hit-and-run, mechanical defects, driving too fast, etc.).

The roadway-related characteristics include the number of vehicle lanes, roadway conditions (e.g. dry, wet, ice, snow, etc.), while the injury involves the time, location, severity and the environment conditions are the weather, lighting conditions, and first harm (e.g. median, fence, pedestrian, etc.)

In order to assess the proposed models, the categorical parameters are quantified and listed in Table 1.

**Table 1:** Summary of the variables.

Variable	Description	Clark County		Washoe	
i) Dependent variables		Proportion (%)			
**Injury severity**	0-PDO	2420(36.3%)		565(35.5%)	
	1-Injury and fatality	4250(63.7%)		1075(65.5%)	
ii) Categorical variables					
**Crash type**	1-Angle	2864(42.94%)		875(53.35%)	
	2-Backing	27(0.40%)		9(0.55%)	
	3-Head-on	82(1.23%)		34(2.07%)	
	4-Rear-end	1999(29.97%)		359(21.89%)	
	5-Sideswipe	241(3.61%)		67(4.09%)	
	6-Non-collision	1452(21.77%)		295(17.99%)	
	0-Unknown	5(0.07%)		1(0.06%)	
**Vehicle 1 type**	1-Car	3638(54.54%)		895(54.57%)	
	2-Truck/bus	979(14.68%)		224(13.66%)	
	3-Motorcycle	1214(18.2%)		296(18.05%)	
	4-Pickup/van	253(3.79%)		161(9.82%)	
	0-Other	586(8.79%)		64(3.90%)	
**Vehicle 1 driver action**	1-Backingup	33(0.49%)		11(0.67%)	
	2-Changing lanes	217(3.25%)		60(3.66%)	
	3-Going straight	3807(57.08%)		810(49.39%)	
	4-Making U-turn	110(1.65%)		25(1.52%)	
	5-Passing other vehicles/racing	30(0.45%)		12(0.73%)	
	6-Stopped	42(0.63%)		8(0.49%)	
	7-Turning left	1211(18.16%)		393(23.96%)	
	8-Turning right	338(5.07%)		78(4.76%)	
	9-Other	81(1.21%)		32(1.95%)	
	10-Unreported	498(7.47%)		102(6.22%)	
	0-Unknown	303(4.54%)		109(6.65%)	
**Vehicle 1 driver condition**	1-Apparently normal	4113(61.66%)		894(54.51%)	
	2-Driving under influence(DUI)	950(14.24%)		324(19.76%)	
	3-Drowsiness, fatigue, fainted etc.	112(1.68%)		21(1.28%)	
	4-Illness/physical impairment	64(0.96%)		8(0.49%)	
	5-Inattentio/distracted	304(4.56%)		63(3.84%)	
	6-Obstructed view	24(0.36%)		3(0.18%)	
	7-Other	191(2.86%)		35(2.13%)	
	0-Unknown	912(13.67%)		292(17.80%)	
**Vehicle 1 condition**	1-Disregarded traffic signs, signals, road markings	688(8.96%)		147(8.97%)	
	2-Driving too fast for conditions	199(2.59%)		43(2.58%)	
	3-Failed to yield right of way	2657(34.64%)		568(34.63%)	
	4-Failure to keep in proper lane	486(6.33%)		104(6.34%)	
	5-Followed too closely	835(10.89%)		179(10.90%)	
	6-Hit and run	112(1.45%)		24(1.44%)	
	7-Made an improper turn	293 (3.81%)		63(3.80%)	
	8-Other improper driving	645(8.41%)		138(8.42%)	
	9-Unsafe backing/lane changing	516 (6.72%)		110(6.73%)	
	10-Other improper driving	670 (8.73%)		143(8.72%)	
	0-Unknown	570 (7.43%)		122(7.44%)	
**Vehicle 2 type**	1-Car	2844(42.64%)		707(43.11%)	
	2-Truck/bus	808(12.11%)		203(12.38%)	
	3-Motorcycle	1196(17.93%)		298(18.17%)	
	4-Pickup/van	1629(24.42%)		363(22.13%)	
	0-Other	193(2.89%)		69(4.21%)	
**Vehicle 2 driver age**	1-less than 18 years old	1832(27.47%)		401(24.45%)	
	2-18- 25years old	987(14.80%)		343(20.91%)	
	3-26-55 years old	3037(45.53%)		725(44.21%)	
	4-56-65years old	513(7.69%)		118(7.20%)	
	0-more than 65 years old	301(4.51%)		53(3.23%)	
**Vehicle 2 driver action**	1-Backingup	4(0.06%)		2(0.12%)	
	2-Changing lanes	30(0.45%)		10(0.61%)	
	3-Going straight	3029(45.41%)		972(59.27%)	
	4-Making U-turn	11(0.16%)		7(0.43%)	
	5-Passing other vehicles/racing	4(0.06%)		0(0.0%)	
	6-Stopped	1584(23.75%)		227(13.84%)	
	7-Turning left	316(4.74%)		45(2.74%)	
	8-Turning right	75(1.12%)		13(0.79%)	
	9-Other	8(0.12%)		1(0.06%)	
	10-Unreported	33(0.49%)		16(0.98%)	
	0-Unknown	1576(23.63%)		347(21.16%)	
**Vehicle 2 driver condition**	1-Apparently normal	4836(72.50%)		1245(75.91%)	
	2-Driving under influence(DUI)	51(0.76%)		17(1.04%)	
	3-Drowsiness, fatigue, fainted etc.	19(0.28%)		0(0.0%)	
	4-Illness/physical impairment	1(0.01%)		0(0.0%)	
	5-Inattentio/distracted	1(0.01%)		0(0.0%)	
	6-Obstructed view	0(0.0%)		0(0.0%)	
	7-Other	6(0.09%)		1(0.06%)	
	0-Unknown	1756(26.33%)		377(22.99%)	
**Vehicle 2 condition**	1-Disregarded traffic signs, signals, road markings	228(8.97%)		35(8.95%)	
	2-Driving too fast for conditions	66(2.58%)		10(2.57%)	
	3-Failed to yield right of way	881(34.63%)		136(34.64%)	
	4-Failure to keep in proper lane	161(6.34%)		25(6.35%)	
	5-Followed too closely	277(10.90%)		43(10.88%)	
	6-Hit and run	37(1.44%)		6(1.40%)	
	7-Made an improper turn	97(3.80%)		15(3.81%)	
	8-Other improper driving	214(8.42%)		33(8.40%)	
	9-Unsafe backing/lane changing	171(6.73%)		26(6.72%)	
	10-Other improper driving	222(8.72%)		34(8.41%)	
	0-Unknown	189(7.44%)		29(7.43%)	
**Road conditions**	1-Dry	6393(95.85%)		1633(99.57%)	
	2-Wet	252(3.78%)		7(0.43%)	
	3-Ice/snow	8(0.12%)		0(0.0%)	
	0-Unknown	17(0.25%)		0(0.0%)	
**Weather conditions**	1-Clear	6613(99.15%)		1583(96.52%)	
	2-Cloudy	26(0.39%)		26(1.59%)	
	3-Rain	3(0.04%)		3(0.18%)	
	4-Other	25(0.37%)		25(1.52%)	
	0-Unknown	3(0.04%)		3(0.18%)	
**Lighting conditions**	1-Daylight	2442(36.61%)		77(4.70%)	
	2-Dark	4043(60.61%)		1550(94.51%)	
	3-Dawn/Dusk	183(2.74%)		11(0.67%)	
	0- Unknown	2(0.03%)		2(0.12%)	
iii) Continuous variables		Mean	Std. Dev.	Mean	Std. Dev.
**TotalVehi**	Total vehicles involved	1.98	0.71	2.00	0.69

### B. DBSCAN crash density analysis

Since DBSCAN was presented by Ester et al. (1996) [41], it has been developing rapidly and considering as one of the mostly cited density-based clustering algorithms in the scientific field today [33]. The main idea of DBSCAN is that all the points within the same cluster are in the range of certain density-reachable radius and density threshold. It is assumed that a dataset *D* contains a set of points p∈*D*, and a density estimates over the data space is required to obtain, thus DBSCAN scans the density around each point with the definition of ϵ-neighborhood.

**Definition 1** ϵ -neighborhood. The ϵ -neighborhood, *N*_ϵ_(p), of a data point p is considered as the set of points within the specified radius ϵ around p, which can be expressed as:

$$N_{\epsilon }=\{q\in D|d(p,q)<\epsilon \}$$ (1)

where *d* denotes the distance measure and ϵ∈ ℝ+, and with q ∈D, this definition indicates that point *p* is always section of its own ϵ-neighborhood, i.e. p∈ *N*_ϵ_(p) always holds. DBSCAN employs *N*_ϵ_(p) and density threshold called *minPts* to identify dense regions and to categorize the points into *core*, *border or noise ones*.

**Definition 2** Point classification. As stated above, point *q*∈*D* is categorized as

- a core point if *N*_ϵ_(p) involves high density, i.e., | *N*_ϵ_(p)| ≥*minPts* where *minPts*∈ ℤ^+^ denotes a user-specified density threshold,
- a border point if *N*_ϵ_(p) is not a core point, but lies in the neighborhood of a core point i.e. *q*∈*D*, i.e. *p*∈ *N*_ϵ_(p), or
- a noise point, otherwise.

**Definition 3** Directly reachable density. A point *q*∈*D* is considered as directly density-reachable from a point *q*∈*D* with ϵ and *minPts* if, and only if | *N*_ϵ_(p)| ≥*minPts* and *q*∈ *N*_ϵ_(p) where p is a core point and q belongs to its ϵ-neighborhood.

**Definition 4** Reachable density. A point *p* is density-reachable from *q* if *D* follows an ordered sequence of points (p_1_, p_2_, …, p_n_) with q = p_1_ and p = p_n_ so that p_i+1_ is directly density-reachable from *pi*∀ *i∈{1*,*2*, *…*, *n− 1}*.

**Definition 5** Density-connected. A point *p*∈*D* is considered as density-connected to a point *q*∈*D* if there exists a point o*∈D* making *p* and *q* density-reachable from *o*.

**Definition 6** Density-based cluster. A density-based cluster *C* is a non-empty subset of D with the followings:

Maximality: If p∈C and *q* is density-reachable from p, then q∈C.

Connectivity: ∀ p, q∈*C*, *p* is density-connected to *q*.

With the definitions abovementioned, DBSCAN algorithm can identify all clusters by searching all core points and density-reachable points with the point *p* and its ϵ -neighborhood. If the point is a core point, a new cluster is formed by clustering all points in the specified radius neighborhood. If another core point is detected in the neighborhood, the same clustering process is performed to involve all points in its neighborhood. The same searching process is continued in the neighborhood till the cluster is finished and the rest points are examined to see whether a new cluster can be formed. The remaining points after all the search process are regarded as noise points.

**Fig 3:**
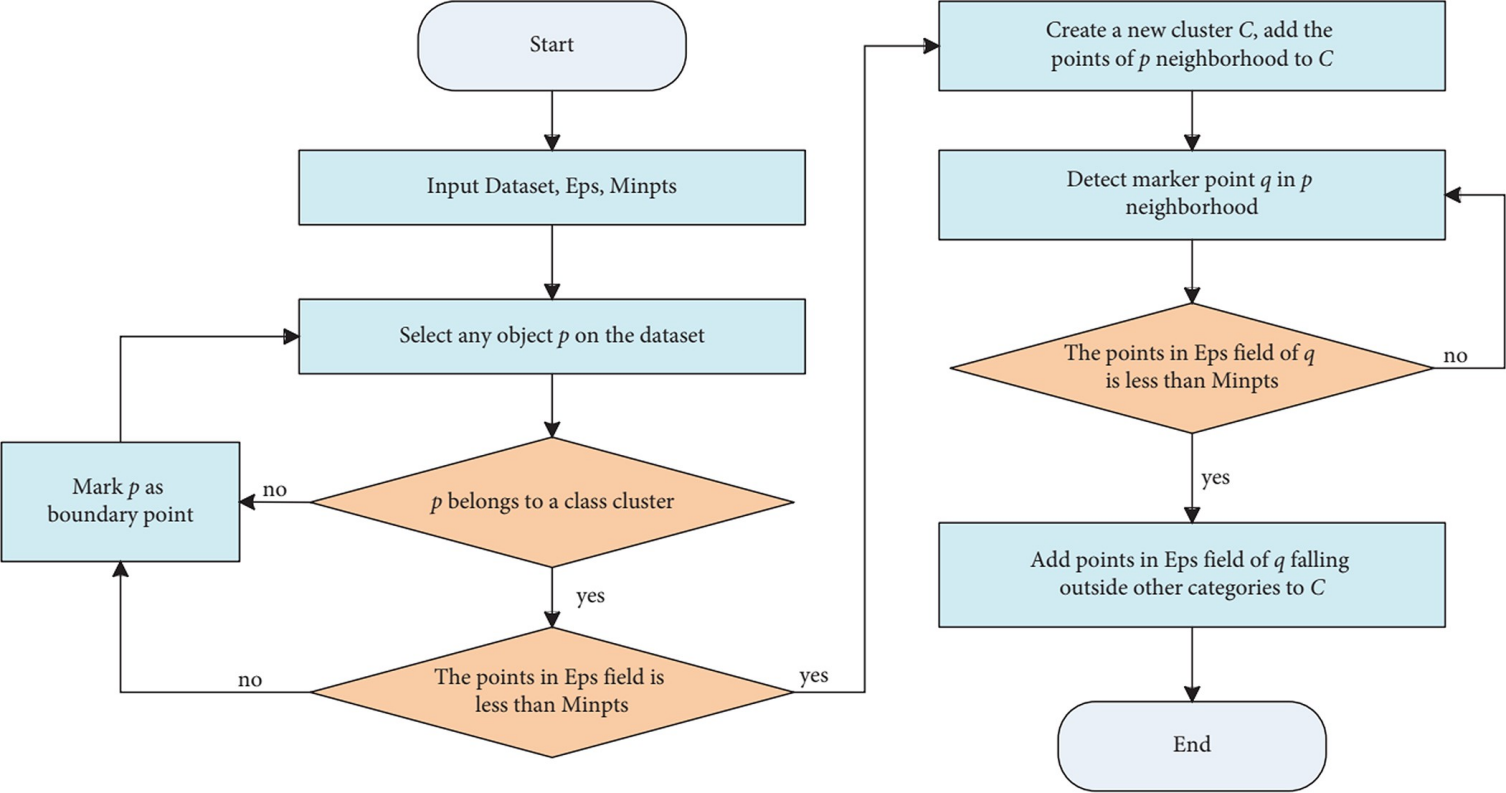
Flowchart of DBSCAN algorithm [42].

The two important parameters ϵ-neighborhood and *minPts* can determine the DBSCAN results, but it’s hard to choose appropriate values because they rely on the dataset selected and influence each other, for instance decreasing *minPts* may lead to decreasing of selected points and vice versa. We will discuss the selection of both parameters in the following section, and high-risk locations can be determined with DBSCAN. Fig 3 gives the flowchart of DBSCAN and more details about DBSCAN can be checked in Reference [33, 42].

### C. Spatial probit model

In order to accommodate the potential within-county correlation or cross-county heterogeneity, the spatial effects are required because neighboring regions typically have identical environmental and geographical characteristics [43]. To this end, spatial binary probit models are employed to analyze the crash data that are concerned with specific locations in space and that denote binary outcomes (e.g. injury severity). Here the spatial probit model with lag version can be expressed as:

$$Y_{i}^{*}=\rho w_{i1}Y_{1}^{*}+\rho w_{i2}Y_{2}^{*}+\cdots +\rho w_{iN}Y_{N}^{*}+X_{i}\beta +\varepsilon_{i}$$ (2)

where $Y_{i}^{*}$ denotes a latent variable, when the observables are binary variables, $Y_{i}=\left\{ \begin{array}{c} 1,if\;Y_{i}^{*}>0\\ 0,if\;Y_{i}^{*}<0 \end{array}\right.$. *ρ* is the dependence parameter to be estimated, *X*_*i*_ is the vector of independent variables, *β* is a parameter vector, *w*_*ij*_ denotes the proximity between observations (*i*, *j*), and *ε*_*i*_ is an i.i.d. random error.

In matrix, Eq (1) can be described as

$$Y^{*}=\rho WY^{*}+X\beta +\varepsilon$$ (3)

where $Y^{*}=(Y_{1}^{*},\cdots ,Y_{N}^{*})\prime$, *W* represents the spatial weight matrices and capture the information of the spatial relationship between observations, and *ε*~ (0, σ^2^*I*_*N*_).

Seen from Eq (3), the model is reduced to the standard probit model if *ρ* = 0, but the estimators employed in the conventional probit model are inconsistent if *ρ*≠ 0; In general, spatial weight matrices are built up as a function of the interval between observations or other contiguity measures. Typically, *w*_*ij*_ = 1 if observations i and j are contiguous, otherwise *w*_*ij*_ = 0. The components of the spatial weight matrix are considered as row-standardization, i.e. ∑_*j*_*w*_*ij*_ = 1, which implies that the spatial lag may be explained as a weighted average of the neighbors; The error terms are heteroskedastic and autocorrelated.

As for the conventional maximum likelihood estimation method, it is not reasonable for limited dependent variable spatial regression models since the spatial likelihood function contains the evaluation of an *n*-dimensional integral, thus the estimation becomes more complicated. Therefore, Bayesian Markov chain Monte Carlo (MCMC) estimation procedure initially proposed by LeSage (2000) [44] is applied, whose process of sampling parameters would not stop until the distribution of draws converges to the predetermined joint posterior distribution of the model parameters. Another reason with Bayesian MCMC lies in that it performs faster than other estimation techniques [45]. Metropolis-Hastings sampler for *ρ* in the spatial lag model is employed and DIC (Deviance Information Criteria) is adopted to assess the model performance. More details about the estimation process can be found in Reference [45–47].

## Results and discussion

In this part, a case study is performed for Clark County and Washoe, respectively. With the case study, the following goals can be obtained: (a) spatial crash pattern for older drivers is identified, (b) a comparative density analysis is performed, (c) high risk locations are presented in terms of visual illustrations, and (d) spatial probit model is conducted to investigate the impact factors of injury severity for older drivers.

### A. GIS-based DBSCAN crash density analysis

As stated in methodology, DBSCAN in GIS is required to input two parameters, -neighborhood () and (*D*). As for each point input, if the number of points within is larger than *D*, this point is considered as core point; if the point is located within, but not core point, it is regarded as border point, otherwise, the point is considered as noise point. The output can be convex or polygon with high-density crash points, and as denoted above, more trials should be conducted before reasonable and *D* are determined. Too large and *D* may cause the high-risk locations to be unidentified, contrarily, too large *p* and too small *D* may produce over-wide high risk locations. Therefore, *R* and *D* have been attempted in the following parts to identify the suitable high-risk locations.

**Fig 4:**
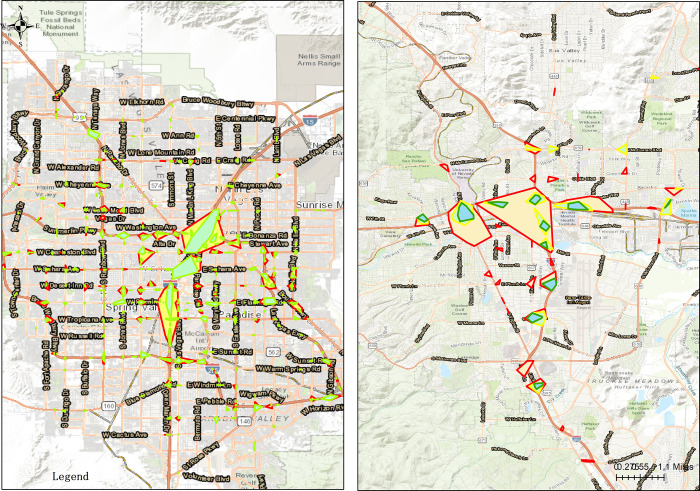
DBSCAN map with R 100ft. (a)Clark County, (b)Washoe.

**Fig 5:**
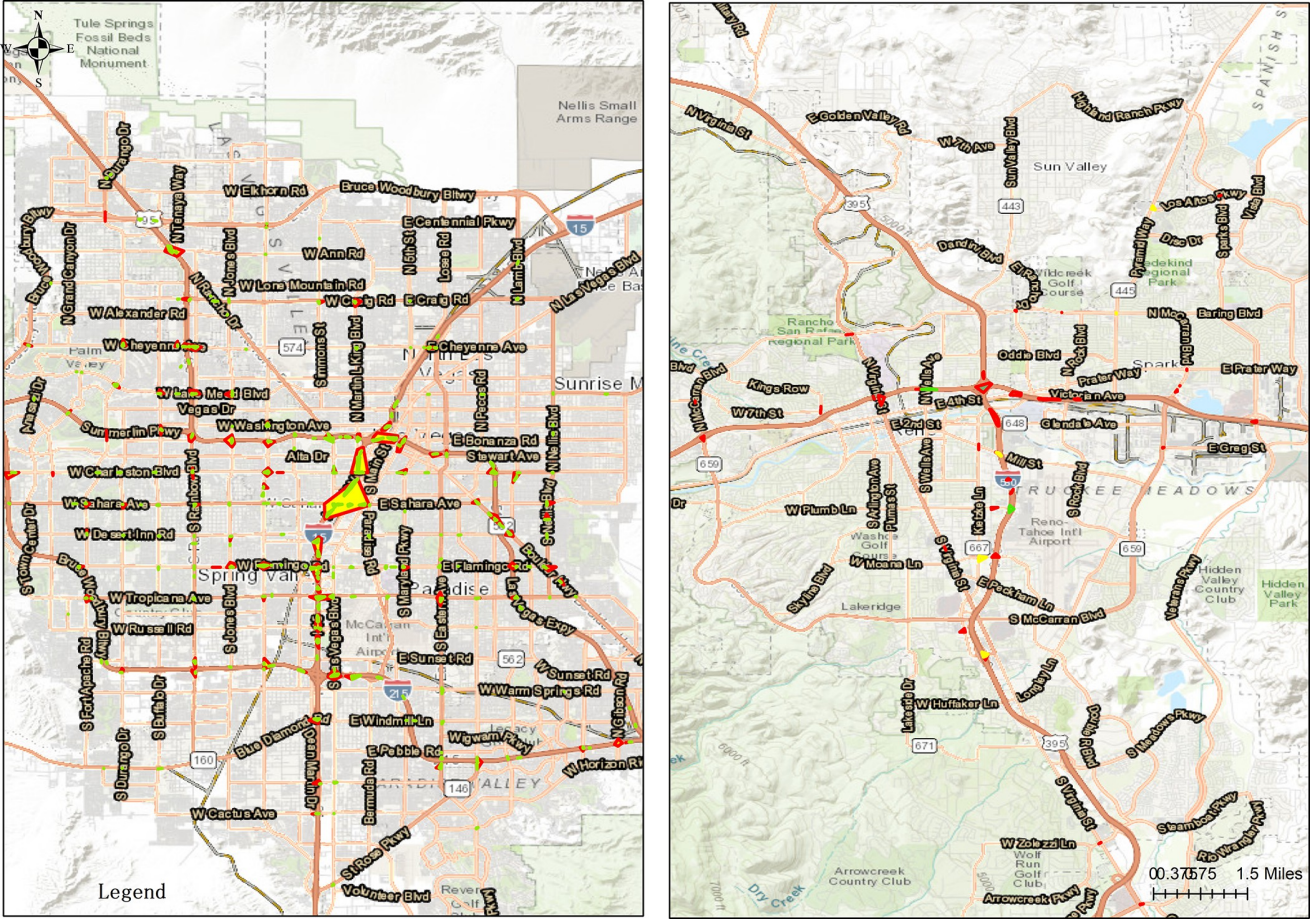
DBSCAN map with R 150ft. (a)Clark County, (b)Washoe.

**Fig 6:**
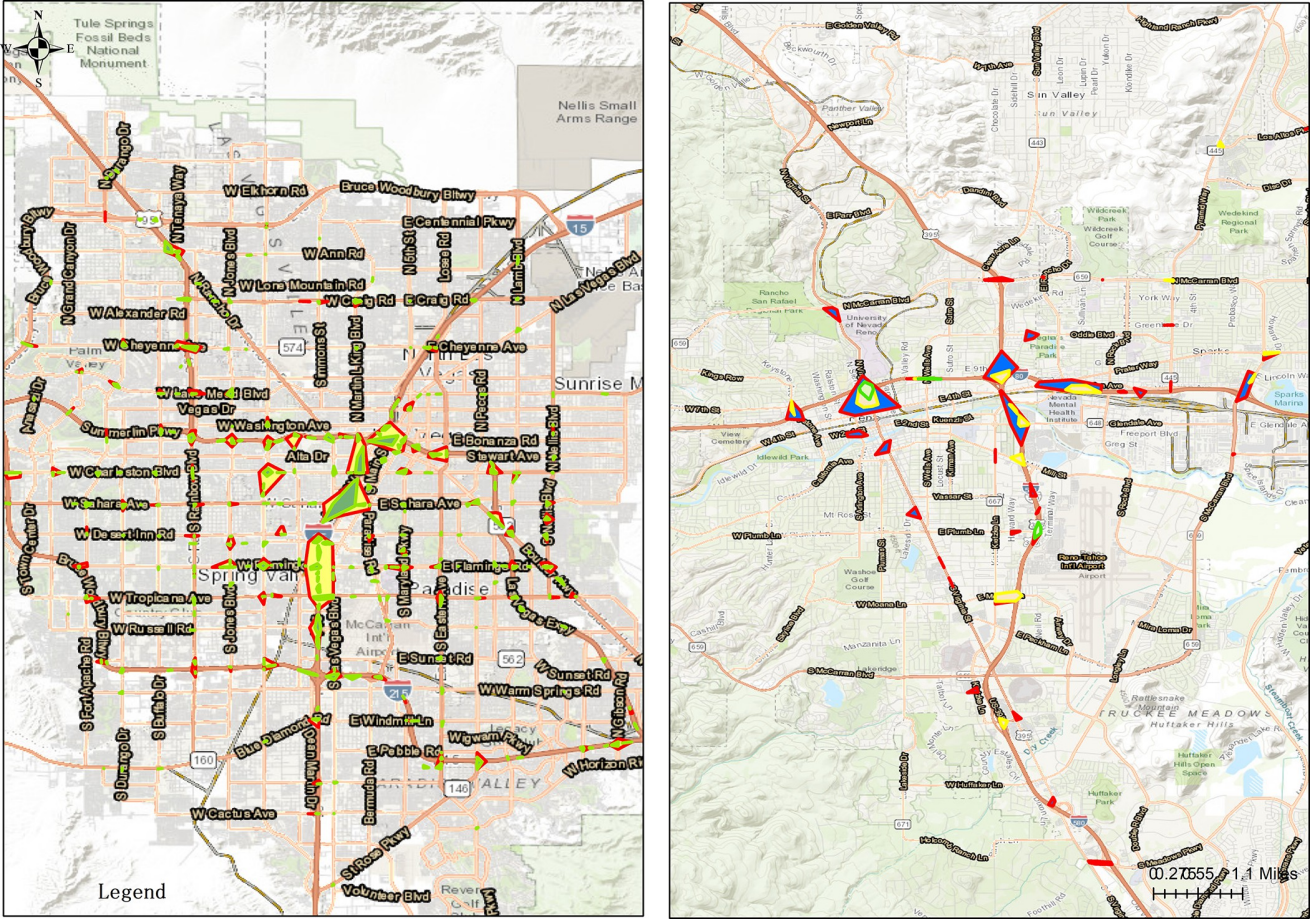
DBSCAN map with R 200ft. (a)Clark County, (b)Washoe.

Figs 4 to 6 give the DBSCAN maps in Clark County and Washoe with *R* 100, 150 and 200 and *D* 5, 10, and 15, respectively. Vertically, in Clark County *R* = 200 and *D* = 15 provide the reasonable number of high density risk locations and density threshold. Concretely, the number of high density risk locations for the three with the same *D* = 5 varies from 259, 289 to 294, indicating that with the increasing of *R*, the number of risk locations are increased, but as a matter of fact, from R = 150 to R = 200 only 5 units are improved, and relatively small compared to the improvement from 100 to 150. Similarly, for the three Rs with the same D = 10, the number of risk locations ranges from 97 to 106 and 116, and varies from 15 to 28 and 36 with D = 15. Fig 6 displays the proper number of high density risk locations and density threshold; In Washoe R = 150 and D = 10 give the suitable number of high density risk locations and density threshold. In detail, the number of high density risk locations for the three with the same *D* = 5 varies from 38, 45 to 50, implying that the number of risk locations increases with R’s increasing, but the rising trend decreases from 7 to 5. Similarly, for the three Rs with the same D = 10, the number of risk locations ranges from 7 to 15 and 16, and varies from 2 to 3 and 11 with D = 15. However, shown from Fig 4 with R = 100 and Fig 6 with R = 200, the convex polygon is way too small or too large, which is beyond the real situation, thus Fig 5 reveals the suitable number of high density risk locations and density threshold.

Horizontally, in Fig 4 with R = 100 for different Ds, the number of high density risk locations and density threshold can’t reveal some trend clearly, while in Fig 5 with R = 150 for different Ds, both the number of high density risk locations and density threshold display certain trend, although the trend in Clark County is still varying. In Fig 6 with R = 200 for different Ds, the number of high density risk locations and density threshold in Clark County gives the reasonable results whereas that in Washoe is way too far from reality.

Consequently, in Clark County DBSCAN map in Fig 6 with R = 200 and D = 15 is selected and the high density risk locations are along Las Vegas Blvd., Boulder Highway, I-215 and several major arterials, e.g. Charleston Blvd., Desert Inn Road and Rainbow Blvd., which mainly reflects the real conditions; In Washoe DBSCAN map in Fig 5 with R = 200 and D = 15 is selected and the high density risk locations lie along I-58, I-90 and interaction with main arterials, e.g. Victoria Ave. and Virginia St., which mainly conforms to the real traffic situation.

### B. Spatial probit model analysis

In this section, a Bayesian spatial probit model was conducted to determine the significant factors affecting the injury severity involving the old drivers, which was compared to the corresponding Bayesian probit model without considering spatial features in Clark County and Washoe separately.

Based on the variables chosen from the four components, the correlation among independent variables is required to examine before running the model. The Pearson correlation test was conducted to avoid the co-linearity. Shown from the test result, vehicle 2 driver condition is highly related to total vehicle and vehicle 2 driver age in Clark County and Washoe, thus, in the final results the three variables may not occur at the same time.

Because the variables go to categorical outcome, multinomial probit regression model was supposed to be the best match, however, the results can’t reflect the spatial features after the model running, and hence spatial probit regression model was turned to. Both the Bayesian binary probit and Bayesian spatial binary probit models were performed to assess the injury severity of older drivers, and to make the comparison and check out whether the non-spatial or spatial model was suitable for this problem. All the insignificant parameters were removed step by step from the model running. Tables 2 and 3 give the results of both models in Clark County and Washoe.

**Table 2:** Results for Bayesian binary probit and Bayesian spatial binary probit models in Clark County.

Variable		Bayesian binary probit				Bayesian spatial binary probit		
	Mean	Std. Dev.	MCSE	95%BCI	Mean	Std. Dev.	MCSE	95%BCI
Crash type	-0.107*	0.008	0.001	(-0.123, -0.089)	-0.106*	0.089	0.001	(-0.383,-0.070)
Vehicle 1 type	-0.062*	0.016	0.002	(-0.091,-0.029)	-0.063*	0.018	0.003	(-0.082,-0.059)
Vehicle 1 action	0.147*	0.006	0.001	(0.003, 0.026)	0.146*	0.008	0.001	(0.002, 0.027)
Vehicle 1 driver condition	0.049*	0.011	0.002	(0.029, 0.074)	0.045*	0.013	0.001	(0.029, 0.073)
Vehicle 1 condition	-0.040*	0.006	0.001	(-0.051, -0.029)	-0.040*	0.007	0.001	(-0.050, -0.029)
Vehicle 2 action	-0.021*	0.007	0.001	(-0.034,-0.007)	-0.020*	0.008	0.001	(-0.034,-0.008)
Vehicle 2 condition	-0.027*	0.011	0.001	(-0.048, -0.006)	-0.028*	0.013	0.001	(-0.049, -0.005)
Road factors	-0.534*	0.042	0.006	(-0.615, -0.452)	-0.534*	0.042	0.006	(-0.615, -0.452)
Weather	-0.054*	0.025	0.003	(-0.103, -0.006)	-0.055*	0.025	0.003	(-0.103, -0.005)
Constant	1.542*	0.043	0.007	(1.455, 1.622)	1.541*	0.044	0.007	(1.456, 1.621)
Marginal Variation					0.813*	0.078	0.005	(0.581, 1.166)
**Goodness-of-fit**								
**No. of observations**			6670			6670		
**Log marginal likelihood**			-4231.500			-4121.413		
**DIC**			8295.919			8043.931		

Note: Std. Dev. = Standard Deviation; MCSE = Monte Carlo Standard Error; BCI = Bayesian credible interval; * denotes significance at 95% confidence interval.

**Table 3:** Results for Bayesian binary probit and Bayesian spatial binary probit models in Washoe.

Variable		Bayesian binary probit				Bayesian spatial binary probit			
	Mean	Std. Dev.	MCSE	95%BCI	Mean	Std. Dev.	MCSE	95%BCI	
Vehicle 1 driver condition	0.058*	0.023	0.001	(0.013, 0.107)	0.055*	0.024	0.001	(0.013,	0.108)
Vehicle 1 condition	-0.048*	0.010	0.001	(-0.070, -0.028)	-0.049*	0.011	0.001	(-0.070, -0.029)	
Vehicle 2 action	-0.051*	0.015	0.001	(-0.082,-0.021)	-0.052*	0.016	0.001	(-0.082,-0.020)	
Vehicle 2 driver condition	0.306*	0.076	0.005	(0.167, 0.462)	0.307*	0.075	0.004	(0.166, 0.463)	
Road factors	-0.419*	0.008	0.004	(-0.582, -0.262)	-0.418*	0.008	0.004	(-0.580, -0.260)	
Constant	0.576*	0.117	0.006	(0.359, 0.807)	0.580*	0.112	0.005	(0.356, 0.810)	
Marginal Variation					0.731*	0.065	0.003	(0.436, 1.043)	
**Goodness-of-fit**									
**No. of observations**			1640			1640			
**Log marginal likelihood**			-1148.508			-759.413			
**DIC**			2211.068			1432.931			

Note: Std. Dev. = Standard Deviation; MCSE = Monte Carlo Standard Error; BCI = Bayesian credible interval; * denotes significance at 95% confidence interval.

Shown from Tables 2 and 3, several observations can be noted. First, the significant parameters of Bayesian binary probit model and Bayesian spatial binary probit model are identical, and the only difference lies in the spatial marginal variation. Second, the standard errors of the mean in the spatial probit model are slightly larger than those in the probit model. Third, the total variation in the spatial binary probit model reveals that there exists spatial dependence, which may lead to estimation bias if being ignored. Fourth, the DIC values (8043.931 and 1432.931) from spatial probit models are much smaller than those (8295.919and 2211.068) from probit models, and the difference is over10, which reveals the models are statistically different. Consequently, the goodness-of-fit of spatial probit model performs better, thus the following enumeration would focus on the Bayesian spatial binary probit model.

Shown from Table 2, crash type is highly associated with injury severity of older drivers. Considered the unknown crash type as the base, the probability of injury severity is decreased with crash type from angle to non-collision, which is uniform with common sense. Among all the crash types, injury due to angle plays an important role for older drivers. It can be calculated that the marginal effect of injury severity probability may be decreased 10.6% if the crash type changes from one to another.

Vehicle type plays an important part in the injury severity of older drivers. Compared to other vehicle types, the probability of injury severity is decreased with vehicle type from truck/bus to car and pickup/van, which makes sense. Generally speaking, the injury severity by truck/bus is more severe than general cars because of the shocking force and size, and some previous studies [48–50] have verified this.

Next, vehicle 1 action is positively related to injury severity of older drivers in Clark County. By comparing to unknown action, the probability of injury severity in increased from “backing up” and “changing lanes” to “turning left” and other actions, which is uniform with general understanding. “Turning left” and other actions of at-fault vehicles may produce more conflicts than “backing up”, thus the chances of running into severe injury is higher, and the marginal effect of injury severity increases 14.6% numerically. Related studies by Guo and Sayed (2020) [51], Wang and Abdel-Aty (2008) [52] provided the similar evidence of injury severity from turning left.

On the contrary, vehicle 2 action is negatively concerned with injury severity of older drivers, i.e. not-at-fault vehicles are exactly opposite to the at-fault vehicles. Since the injury is caused by both sides, it is reasonable that at-fault vehicles action leads to the increasing probability of injury severity, while not-at-fault vehicles actions generates the decreasing probability.

Another significant parameter, the vehicle 1 driver condition (i.e. at-fault driver condition) is positively related to injury severity of older drivers, which indicates that when the vehicle drivers’ conditions vary from “apparently normal” to “obstructed view” and “other conditions”, the severity is getting worse, and the marginal effect of probability is increased 4.5% during this variation. This indicates that at-fault older drivers’ conditions indeed play very important roles in the injury.

Vehicle conditions reveal negative relation with injury severity of older drivers. Whether at-fault or not-at-fault vehicles, the variation from “disregarded traffic signs, signals, road markings” and “driving too fast for conditions” to and “other improper driving” reduces the severe injury, which reflects that distraction and speeding are more likely to cause injury severity. Similar studies by Abegaz et al. (2014) [53] and Donmez and Liu (2015) [54] reveal the identical results.

The last two significant variables road factors and weather are negatively concerned with injury severity of older drivers. Compared to unknown road factors, most crash injuries occur under the dry roadway conditions while wet/ice/snow road factors lead to severe injury, which is understandable in real life. Similarly, most crash injuries happen in clear weather whereas severe injuries often result from rainy or adverse weather conditions. Various studies [55, 56] have verified these.

As for injury severity in Washoe of Table 3, the vehicle drivers’ conditions, whether at-fault driver or not-at-fault driver, are positively related to injury severity of older drivers, which displays that when the vehicle drivers’ conditions vary from “apparently normal” to “obstructed view”, the severity is getting worse, and the marginal effect of possibilities are increased 5.5% and 30.7% during this variation, respectively. This represents the injury severity escalates higher for not-at-fault older drivers’ conditions than at-fault drivers’ conditions.

Similar to results in Clark County of Table 2, vehicle 1 condition gives negative relation with injury severity of older drivers in Washoe. As for at-fault vehicle condition, the changing from one type to another reduces the marginal effect of severe injury possibility by about 4.9%, which indicates that distraction and speeding are more likely to produce injury severity. Similarly, road factors are negatively related to injury severity of older drivers. This implies that road factors play the similar role in Washoe as in Clark County. Different from Clark County, the weather conditions are not significant, which indicates in Washoe the weather changing doesn’t have much impact on injury severity of older drivers.

Shown from Tables 2 and 3, the closer observation of the estimated results displays some similarities and differences between the two locations. First, the similarity lies in that among all the influencing parameters, vehicle 1 driver condition, vehicle 1 condition, vehicle 2 action and road factors are of significance for injury severity of older drivers in two locations. This indicates that injury severity influenced by driver condition and vehicle condition. Second, the difference lies in that significant parameters in Washoe are fewer than those in Clark County. This implies that the factor sources of injury severity in Clark County come from more aspects than those in Washoe.

According to the results above, empirically, first, at the high density risk locations in Clark County and Washoe, countermeasures should be taken to alert older drivers to prevent the vehicles from confliction, and older drivers should pay more attention to the pedestrians, electronic devices, traffic signs, labels and marks; Then, older drivers with heavy vehicles should be limited since in the sensory, cognitive and decision-making abilities they decline and sometimes are slow in maneuver control, which might cause severe injury; Next, under adverse weather conditions, e.g. fog, rainy or snow day, older drivers should be alert and pay more attention to pedestrians and other automobiles; At last, more physical examinations should be taken regularly to guarantee the drivers’ conditions are kept healthy.

## Conclusions

With the ageing society approaching, older driver group, as one of the most vulnerable and highest risk roadway users, takes much pressure in influencing factors of injury severity. In this study DBSCAN approach was presented to perform the spatial analysis in two urban counties in Clark County and Washoe, Nevada, and Bayesian spatial probit model was proposed to determine the significant impact factors of injury severity for older drivers. Four main goals were obtained: (a) spatial crash pattern for older drivers was identified in Clark County and Washoe respectively; (b) a comparative density analysis was conducted with different density radiuses by DBSCAN;(c) high risk locations were presented in terms of geo-visualization with ArcGIS; and (d) spatial probit models were performed to determine significant influencing factors of injury severity for older drivers. Consequently, the spatial crash pattern and density analysis for older drivers can be determined with DBSCAN, as well as high risk locations in terms of geo-visualization, which provides a solid option for the spatial analysis, and hotspot identification of injury severity over current studies. Furthermore, Bayesian spatial binary probit model addresses the factor determinants spatially, expanding the geo-statistical analysis scope, and extending into a new level of injury severity.

As abovementioned, there have been different methods and approaches about the injury severity analysis of older drivers. However, most of the studies focus on the hotspots or impact factors generally and individually, and there may exist spatial properties of injury severity. In this study, in order to account for the potential within-county correlation or cross-county heterogeneity, the DBSCAN and Bayesian spatial probit model are proposed, which can address the spatial properties of injury severity in two locations about older drivers, and accommodate the significant impact factors of injury severity, which provides the support for two main findings the work. First, DBSCAN approach provides the spatial crash pattern for older drivers, and determines high risk spots of injury severity in different locations. Second, Bayesian spatial binary probit model identifies the significant influencing factors spatially, which extends the range of injury severity analysis.

Some weakness may need to be strengthened in the future study. More variables related to older drivers’ characteristics are required to collect, such as driver’s personal status, physical and psychological status, education level, driving habits (passive or aggressive) etc., and with those variables the older drivers’ injury severity can be reflected completely. Another problem is that the results are based on the dataset from Nevada, and it is worthy of utilizing different data sources to confirm the findings and transferability in the future. Future study may need to consider the infrastructure of ageing society on the roadway, vehicles and environment for the elders, so that more specific countermeasures can be presented to improve the elders’ health and care.
